# Effect of the +781C/T Polymorphism in the Interleukin-8 Gene on Atherosclerotic Cerebral Infarction, and Its Interaction with Smoking and Drinking

**DOI:** 10.1371/journal.pone.0080246

**Published:** 2013-11-07

**Authors:** Shijian Luo, Feng Wang, Zhendong Li, Jinfeng Deng

**Affiliations:** Department of Neurology, The Fifth Affiliated Hospital of Sun Yat-sen University, Zhuhai, Guangdong, China; National Central University, Taiwan

## Abstract

**Objective:**

The aims of this study were to investigate the association between the +781C/T polymorphism of interleukin-8 (IL-8) and atherosclerotic cerebral infarction and the interaction between the +781C/T polymorphism and smoking or drinking in cerebral infarction in the Han Chinese population.

**Methods:**

We investigated the +781C/T polymorphism of *IL-8* in 308 consecutive Han Chinese patients who were diagnosed with atherosclerotic cerebral infarction and in 294 age- and gender-matched healthy control subjects. The patients were classified using the Oxfordshire Community Stroke Project (OCSP) classification. The patients and subjects’ histories of smoking and drinking were recorded, and atherosclerosis (AS) of the internal carotid artery (ICA) was evaluated in the patients. The +781C/T polymorphism was determined by polymerase chain reaction and restriction fragment length polymorphism (PCR-RFLP) analysis.

**Results:**

The +781C/T polymorphism and allele frequencies were not significantly different between the patients and controls and were not significantly associated with the OCSP classifications. We found that the 781C allele was significantly associated with AS of the ICA in the patients (*p* = 0.017), and the CT genotype was more prevalent in patients without AS of the ICA (*p* = 0.035). No interactions were observed between the +781C/T polymorphism and smoking or drinking.

**Conclusion:**

Our results demonstrated that the +781C/T polymorphism of *IL-8* did not play a role and had no interaction with smoking or drinking in the occurrence of cerebral infarction in the Han Chinese population. However, the C allele and the CT genotype might be associated with AS of the ICA in patients with ischemic stroke.

## Introduction

Cerebrovascular diseases have become the most common cause of death in China [1]. Atherosclerotic cerebral infarction is the most common form of stroke. Many studies have indicated that inflammation plays an important role in the formation of atherosclerosis and ischemic brain injury. Interleukin-8 (IL-8) is a typical member of the CXC chemokine subfamily and a strong chemoattractant factor for neutrophils, basophilic leukocytes and T lymphocytes via firm binding to its receptors, CXCR1 or CXCR2. As a strong mediator of inflammation, IL-8 plays an important role in promoting the formation of atherosclerosis (AS) [2] and is involved in the process of brain injury after acute cerebral infarction [3–5]. Many studies have indicated that genetic polymorphisms were associated with the occurrence of cerebral infarction [6]. Some *IL-8* single nucleotide polymorphisms (SNPs) including +781C/T (rs2227306) were confirmed to be related to the transcriptional level of *IL-8* [7], and may be associated with the occurrence or development of a variety of diseases [8–12]. Furthermore, a previous study suggested that the +781C/T polymorphism might play a more important role than other SNPs of *IL-8*, such as -251A/T (rs4073), 1633C/T and 2767A/T, in the transcriptional level of *IL-8*, which associated strongly with asthma occurrence [13].

Currently, to our knowledge, there have been only three association studies of *IL-8* polymorphisms and cerebral infarction. Grau et al. [14] revealed that the 250T/A polymorphism in the promoter of *IL-8* did not correlate with IL-8 release in young adults with ischemic stroke. Enquobahrie et al. [15] reported that three SNPs, rs4073, rs2227307 and rs2227543, were not associated with the risk of ischemic stroke. Kis et al. [16] detected that the serum IL-8 levels of the patients with non-cardiogenic stroke were significantly higher than those in the healthy controls but did not reveal that the -251A/T polymorphism, located in promoter region of *IL-8*, was correlated with the occurrence of cerebral infarction. However, the association between the polymorphism of +781C/T and the occurrence of atherosclerotic cerebral infarction is unclear. In addition, smoking and drinking are the common environmental risk factors for AS and cerebral infarction. Some epigenetic studies revealed that the interactions between certain gene polymorphisms and smoking or alcohol consumption on the risk of cerebral infarction [17–24]. However, whether the +781C/T polymorphism interacts with smoking or drinking is unclear.

The aims of this study were to examine the association between the +781C/T polymorphism in *IL-8* and atherosclerotic cerebral infarction and to investigate the interaction between the +781C/T polymorphism and smoking or drinking in cerebral infarction in the Han Chinese population.

## Materials and Methods

### Cases and controls

A total of 308 unrelated consecutive Han Chinese patients with the first onset of atherosclerotic cerebral infarction who were hospitalized in 2010 and 2011 were selected for study at the Fifth Affiliated Hospital of Sun Yat-sen University, as previously described [25]. They met the WHO diagnostic criteria for cerebral infarction [26]. Patient diagnoses were also verified with either a CT or an MRI. The evidence for AS in the patients included eyeground AS and internal carotid artery (ICA) intima-media wall thickness (IMT) greater than 10 mm verified by color Doppler ultrasonography, and cerebral vascular AS was verified by CTA or DSA. Those patients with non-atherosclerotic cerebral infarctions, hemorrhagic stroke, blood diseases, malignant tumors, autoimmune diseases, inflammatory diseases and a history of ischemic cerebrovascular disease were excluded from the study. The controls comprised 294 age- and gender-matched subjects, who were Han Chinese volunteers recruited from the hospital (for routine health visits). All participants gave their written informed consent. This study was also approved by the Independent Ethics Committee of the Fifth Affiliated Hospital of Sun Yat-sen University. To investigate the interaction between the +781C/T polymorphism and smoking or drinking, we performed the case-only analysis with the patients.

### Clinical evaluation

The medical history of the patients and controls, such as age, sex, birthplace, nationality, history of smoking (≥ 1 cigarette/d for at least one year or ≥ 360 cigarettes per year) [22], drinking (≥ 15 g/d of ethanol) [27], hypertension and diabetes, were recorded. Examinations, such as routine blood tests, erythrocyte sedimentation rate, blood glucose level, blood lipid level, homocysteic acid level, electrocardiogram, chest direct digital radiography, color Doppler ultrasonography of the carotid arteries and heart, head CT/CTA and head MRI/MRA, were performed in both groups. All patients were classified into four groups using the Oxfordshire Community Stroke Project (OCSP) classification [28]: total anterior circulation infarction (TACI), partial anterior circulation infarction (PACI), posterior circulation infarction (POCI) and lacunar cerebral infarction (LACI).

### Genetic analyses

**Figure 1:**
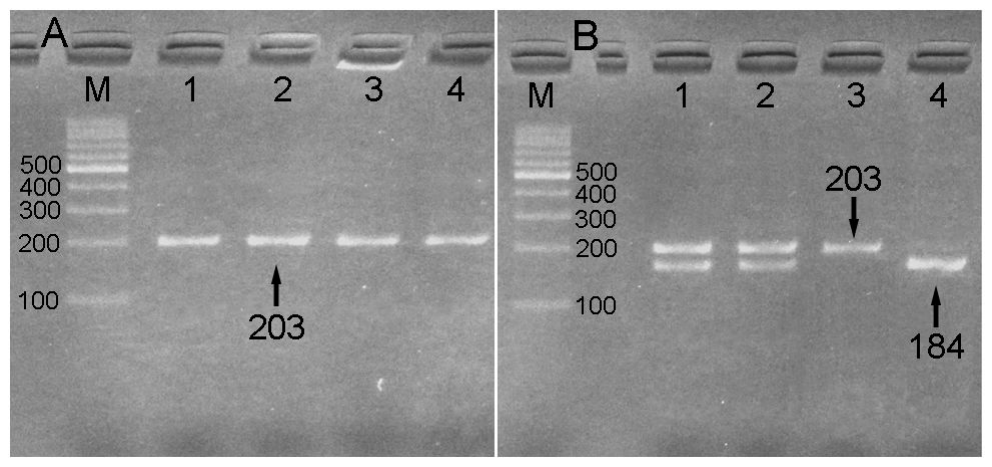
Electrophoresis of PCR products of the samples and genotyping of the *IL*-8 +781C/T SNP. (A) *IL*-8 +781C/T SNP: lane M, 100 bp marker ladder, lanes 1–4, samples, the 203 bp bands are the PCR products. (B) genotyping of the *IL*-8 +781C/T SNP: lane M, 100 bp marker ladder; lanes 1 and 2, CT genotype (203-, 189- and 19-bp); lanes 3, TT genotype (203 bp); and lanes 4, CC genotype (184- and 19-bp). The 19 bp fragment was invisible in the gel owing to its fast migration speed.

Genomic DNA was prepared for genetic analysis from EDTA.K_2_ anticoagulated peripheral blood with the E.Z.N.A.™ SQ DNA Kit II (Omega Bio-Tek, Inc. Norcross, GA, USA), as previously described [25]. To analyze the +781C/T polymorphism of *IL-8*, PCR-RFLP was performed. Amplification of a 203-bp fragment of *IL-8* was performed in a total volume of 25 μl with 300 ng of extracted genomic DNA, 12.5 μl of 2X Taq Master Mix (Omega Bio-Tek, Inc. Norcross, GA, USA) and the two primers. The forward primer was 5'-CTCTAACTCTTTATATAGGAATT-3' and the reverse primer was 5'-GATTGATTTTATCAACAGGCA-3' [13]. The PCR consisted of 1 cycle of 5 min at 94°C; 35 cycles of 30 s at 94°C, 30 s at 50°C and 60 s at 72°C; and 1 cycle of 10 min at 72°C in a DNA Engine Option^TM^ 2 PCR (MJ Research Corporation. Watertown, Mass., USA). The PCR product (10 μl) was cleaved at 37°C with a 5 U EcoRI restriction enzyme (Fermentas, Canada) for approximately 4 hours. To analyze the +781C/T polymorphism, EcoRI digestion of the PCR product yielded bands of 184 bp and 19 bp in the CC homozygotes, 203 bp in the TT homozygotes and all bands (203 bp, 184 bp and 19 bp) in the CT heterozygotes (Figure 1).

### Statistical analysis

All statistical analyses were performed with the SPSS 13.0 statistical package (SPSS Inc., Chicago, IL, USA). Differences between groups for continuous values were calculated by Student’s *t*-test. For dichotomous variables, chi-square (χ^2^) analysis or Fisher's exact test and logistic regression analyses were applied. The criterion for statistical significance was a *p-value* of < 0.05.

## Results

The participants were from 28 provinces of China, and no significant difference was found regarding the birthplace between the patients and controls (*p* = 0.327), as previously described [25]. The two groups were well-matched in terms of age and sex. The patients had smoking, drinking, hypertension, diabetes mellitus, high homocysteine levels and AS of the ICA more often than the controls (Table 1).

**Table 1:** Population characteristics.

	**Patients**	**Controls**	***P*^a^**
N	308	294	
Age, y (mean ± SD)	63.63 ± 13.02	61.72 ± 11.58	0.058
Male (n)	205	183	0.269
Smoking (n)	122	58	<0.001
Drinking (n)	50	25	0.003
Hypertension (n)	233	105	<0.001
Diabetes mellitus (n)	99	39	<0.001
Hyperlipoidemia (n)	159	154	0.385
High homocysteine (n)	84	33	<0.001
AS in ICA (n)	224	160	<0.001

a Chi-square test was used to compare values of all parameters in patients and controls except for age, which was compared by Student's *t*-test.

The +781C/T genotypes of *IL-8* were successfully analyzed in all participants. The genotype and allele frequency distributions among the patients and controls are compared in Table 2 and were consistent with those expected from Hardy-Weinberg equilibrium (*p* =0.297).

**Table 2:** Association of 781C/T polymorphism with cerebral infarction or OCSP classification.

				**OCSP classifiction**				
	**Patients (n = 308)**	**Controls (n = 294)**	***P***	**TACI (n = 37)**	**PACI (n = 160)**	**POCI (n = 47)**	**LACI (n = 64)**	***P***
**Genotypes**								
CC	140 (0.454)	123 (0.418)		12 (0.324)	73 (0.456)	23 (0.489)	32 (0.500)	
CT	128 (0.416)	133 (0.452)		21 (0.568)	66 (0.413)	21 (0.447)	20 (0.313)	
TT	40 (0.130)	38 (0.130)	0.631	4 (0.108)	21 (0.131)	3 (0.064)	12 (0.187)	0.176
**Allele frequencies**								
C	408 (0.662)	379 (0.645)		45 (0.608)	212 (0.663)	67 (0.713)	84 (0.656)	
T	208 (0.338)	209 (0.355)	0.517	29 (0.392)	108 (0.337)	27 (0.287)	44 (0.344)	0.559

Chi-square test was used to compare values of all parameters.

The +781C/T genotype distribution and allele frequencies were not significantly different between the patients and controls. In addition, neither the genotype distribution nor the allele frequencies of +781C/T were significantly associated with the OCSP patient classifications (Table 2).

Color Doppler ultrasonography of the carotid arteries were performed in 283 patients. There were 224 patients (male 154, female 70, age 60.76±11.56) with AS of the ICA and 59 patients (male 36, female 23, age 59.32±12.31) without AS of the ICA. The two subgroups (with or without AS in the ICA) were well-matched in terms of age (*p* = 0.074) and sex (*p* = 0.261). The +781C/T genotype distribution and allele frequencies were significantly different between the two subgroups (Table 3). Adjustments for the patients’ conventional risk factors (smoking, drinking, hypertension, diabetes mellitus, hyperlipidemia, high homocysteine) were performed by including the +781C/T genotypes and alleles in a logistic regression model. The 781C allele was significantly associated with AS of the ICA in the patients (*p* = 0.017, *OR* 1.657, 95% CI 1.094 - 2.510). The CT genotype was more prevalent in the patients without AS of the ICA than in the patients with AS of the ICA (*p* = 0.035, *OR* 0.474, 95% CI 0.237 - 0.949). 

**Table 3:** Association of 781C/T polymorphism with AS of ICA in patients.

	**Patients**			
	**AS in ICA**	**Non-AS in ICA**	***OR (95%CI)***	***P*^a^**
N	224	59		
Age, y (mean ± SD)	60.76 ± 11.56	59.32 ± 12.31		0.074
Male (n)	154	36		0.261
Genotype				
CC	111 (0.496)	18 (0.305)		0.095
CT	85 (0.379)	31 (0.525)	0.474（0.237-0.949）	0.035
TT	28 (0.125)	10 (0.170)	0.514（0.200-1.322）	0.167
Allele frequencies				
C	307 (0.685)	67 (0.568)		
T	141 (0.315)	51 (0.432)	1.657(1.094-2.510)	0.017

a Logistic regression analysis was used to compare values of genotype and allele frequencies. Age was compared by Student's *t*-test and male was compared by Chi-square test.

We found that the +781C/T polymorphism did not significantly interact with smoking or drinking (Table 4).

**Table 4:** Interaction between 781C/T polymorphism of IL-8 and smoking or drinking in patients.

	**Patients**				**Patients**			
**Genotype**	**Smoking(n = 122)**	**No-smoking(n = 186)**	***OR* (*95% CI*)**	***P***	**Drinking(n = 50)**	**No-drinking(n = 258)**	***OR* (*95% CI*)**	***P***
CC	57 (0.467)	83 (0.446)			23 (0.460)	117 (0.453)		
CT and TT	65 (0.533)	103 (0.554)	0.919 (0.581 - 1.453)	0.718	27 (0.540)	141 (0.547)	0.974 (0.530 - 1.789)	0.933

Logistic regression analysis was used to compare values of all parameters.

## Discussion

The IL-8 mRNA levels expressed in peripheral blood mononuclear cells (PBMC) has been shown to increase rapidly during the acute stage of cerebral infarction [29]. The IL-8 concentrations also increased rapidly in the plasma and CSF of patients with acute ischemic stroke [14,29–31]. Currently, there have been few association studies between the SNPs of *IL-8* and cerebral infarction. No previous study has investigated the association of the +781C/T polymorphism with atherosclerotic cerebral infarction, especially in the Han Chinese population. Our study revealed that the +781C/T polymorphism in *IL-8* was not associated with atherosclerotic cerebral infarction. Taken together, this result and the findings of other studies might demonstrate that the common SNPs of *IL-8* have no associations with cerebral infarction [15,16]. Atherosclerotic cerebral infarction is a disease caused by a combination of multiple factors and genes, and thus the effects produced by one or several SNPs on the occurrence of diseases are very slight and the risk of an individual suffering from cerebral infarction may be determined by the synergy of some risk factors. Whether the +781C/T polymorphism of *IL-8* affects the occurrence and development of cerebral infarction by interacting with other genes requires further research. The OCSP classification of cerebral infarction revealed that brain areas were damaged by the obstruction of certain corresponding feeding arteries after ischemic stroke [28]. IL-8 is involved in the process of brain injury after atherosclerotic cerebral infarction, but the contributions of the +781C/T genotype and allele frequencies to the OCSP subtypes were not significantly different in this study. We speculate that the +781C/T polymorphism of *IL-8* did not selectively affect the cerebrovasculature.

Many studies indicated that the variants of some genes could promote the occurrence and development of AS in the ICA, such as the genes encoding inflammatory cytokines, *angiotensinogen*, *matrix metalloproteinase-3* and *endothelial nitric oxide synthase*, which can induce cardiovascular diseases and cerebrovascular diseases [32–35]. Rundek et al. [36] found that the polymorphisms of *interleukin-6* were associated with AS of the ICA and that GG homozygous individuals had a high likelihood of developing AS of the ICA. Currently, no previous study has investigated the +781C/T polymorphism with respect to AS of the ICA in the Han Chinese population. In this study, we found that the 781C allele frequency was significantly higher in the patients with AS of the ICA than in the patients without AS of the ICA and that the CT genotype was more common in the patients without AS of the ICA. These results indicated that the 781C allele was a risk factor for AS of the ICA and the CT genotype might protect individuals suffering from AS of the ICA. We also can reduce the occurrence of AS by interfering with this variant of +781C/T. However, this study only revealed that the +781C/T polymorphism of *IL-8* was associated with AS of the ICA in the patients with atherosclerotic cerebral infarction in the Han Chinese population because the subjects of the study were all patients with stroke. Further special investigations are needed to determine whether other SNPs of *IL-8* also play particular roles in AS of the ICA or whether the +781C/T polymorphism interacts with other SNPs of *IL-8* to affect AS of the ICA.

Atherosclerotic cerebral infarction is a complex process with roles played by multiple factors and a multi-stage development in which the factors of environment and heredity are involved. Atherosclerosis is the most common cause of cerebral infarction. Smoking and drinking may accelerate the development of AS, finally leading to arteriostenosis and thrombogenesis. Currently, some studies have reported about the interaction between gene polymorphisms and smoking or drinking with respect to the occurrence and development of cerebral infarction [17–24]. For examples, one study had shown that smokers with the D allele of *angiotensin-converting enzyme* had a higher susceptibility to ischemic stroke than those with the I allele of *angiotensin-converting enzyme* [22]. Another study indicated that the risk of ischemic stroke increased significantly in smoking individuals with the T allele of *N* ^*5*^ *, N* ^*10*^ *-methylene tetrahydrofolate reductase* than in those with the C allele [23]. Our study revealed that these interactions between the +781C/T polymorphism and smoking or drinking might not result in synergistic effects on the occurrence of cerebral infarction. But smoking and drinking might affect the occurrence of cerebral infarction by other ways, especially abnormal DNA methylation [37–39].

The sample sizes of the existing similar studies in the Han Chinese population were not larger than 200 patients, which might affect the reliability of the results [3,22]. The results of our study might be more reliable because we finished this study with a larger sample size and used a mature detection technology. In particular, we excluded miscellaneous factors in our study, such as autoimmune diseases, malignant tumors, and inflammatory diseases. We did not evaluate other variants of *IL-8* in this study, and thus the interactive effects of these SNPs on cerebral infarction could not be determined. However, these findings represent significant results in the Han Chinese population.

In conclusion, our findings indicated that the +781C/T polymorphism of *IL-8* did not play a role and had no interaction with smoking or drinking in the occurrence of cerebral infarction in the Han Chinese population. However, the C allele and the CT genotype might be associated with AS of the ICA in those patients with ischemic stroke.
